# The first whole-genome sequence of a prospective novel sponge-associated *Streptomyces *strain from Indonesia with a long 1.5 Mbp terminal inverted repeat

**DOI:** 10.12688/f1000research.173356.2

**Published:** 2026-01-16

**Authors:** Rahadian Pratama, Linda Sukmarini, Akhirta Atikana, Shanti Ratnakomala, Fahrurrozi Fahrurrozi, Puspita Lisdiyanti

**Affiliations:** 1 Department of Biochemistry, IPB University, Bogor, West Java, Indonesia; 2 Research Centre for Applied Microbiology, National Research and Innovation Agency (BRIN), Bogor, West Java, Indonesia; 3 Indonesian Biofilm Research and Collaboration Centre, Yogyakarta, Yogyakarta, Indonesia; 4 Laboratory of Microbiology, Wageningen University & Research, Wageningen, Gelderland, The Netherlands; 5 Research Centre for Biosystematics and Evolution, National Research and Innovation Agency (BRIN), Bogor, West Java, Indonesia; 6 Research Centre for Freshwater, National Research and Innovation Agency (BRIN), Bogor, West Java, Indonesia

**Keywords:** Melophlus sarasinorum, sponge-associated Streptomyces, de novo assembly, whole genome, L-TIR

## Abstract

*Streptomyces* sp. BTA 1-131 was isolated from the marine sponge *Melophlus sarasinorum* collected in Indonesia. The crude extracts of this strain displayed antibacterial and cytotoxic activity, and therefore, to further investigate the bioactive potential of the strain, whole genome sequencing was performed in this study. The whole genome sequencing of *Streptomyces* sp. BTA 1-131 was conducted using both Illumina NextSeq and Oxford Nanopore platforms with a de novo hybrid assembly approach. The high-quality genome obtained is 10.23 Mbp with a GC content of 71.57%. It is organised into a single chromosomal contig, two linear plasmids, and one circular plasmid. Interestingly, a long-terminal inverted repeat (L-TIR) sequence of 1.5 Mbp has been confirmed in the strain genome. Phylogenomic analysis suggested that the strain BTA 1-131 likely represents a new species within the genus *Streptomyces.* To the best of our knowledge, the genome data described here would be the first report on the hybrid genome sequence of *Streptomyces* associated with the rarely reported sponge *Melophlus sarasinorum* from Indonesia, with a unique feature of L-TIR. The complete genome data generated here will provide compelling information for further analysis of the biosynthetic potential of the strain BTA 1-131 to produce new bioactive compounds.

## Introduction

Streptomycetes represent a group of metabolically diverse bacteria known for their ability to produce natural products of considerable importance in human health, veterinary medicine, and agriculture. The genus *Streptomyces* primarily synthesises bioactive secondary metabolites that include antibiotics, as well as antifungal, antiviral, and anthelmintic agents, anticancer drugs, immunosuppressants, and herbicides. ^1^ However, *Streptomyces* spp. demonstrate notable GC content exceeding 70% and show high repetition within secondary metabolite biosynthetic gene clusters (BGCs). ^2^ These provide issues in sequencing and high-quality genome assembly for in-depth genome mining, which are presently addressed through intensive sequencing efforts. Advances in genome sequencing technology have uncovered a multitude of hitherto unexamined metabolic capabilities inside *Streptomyces* genomes. The advent of fast and efficient genome sequencing tools has allowed for the examination of more profound and comprehensive bacterial genomic data. Thus, the data rely on the thoroughly curated genome sequence characterised by high contiguity and improved annotation. In recent years, the emergence of long-read sequencing technologies has led to the integration of both Illumina (short) and Nanopore or PacBio (long) reads, potentially enhancing the completeness of genome assemblies relative to those constructed based solely on Illumina reads. ^3^

*Streptomyces* sp. BTA 1-131 was isolated from an Indonesian sponge, namely MS5, identified as *Melophlus sarasinorum.* The marine sponge *M. sarasinorum* is widely distributed in the Indo-Pacific region, including Indonesia, Palau, Guam, Papua New Guinea, and the Solomon Islands. ^4^ A multiomics analysis of *M. sarasinorum* leads to the exploration of the previously unknown natural products derived from the sponge *M. sarasinorum* and the bacterial symbiont. ^5^ Indeed, our initial study showed the potential extracts of sponge *M. sarasinorum* MS5 to inhibit the growth of gram-positive pathogenic bacteria, and the abundance of actinobacteria is significantly correlated to the sponge bioactivity. ^6^ Further investigation observed antibacterial activity in the methanolic extracts of the sponge-associated actinobacterium *Streptomyces* sp. BTA 1-131 against the gram-positive *S. aureus* ATCC 13240. Additionally, the methanolic extracts of *Streptomyces* sp. BTA 1-131 also demonstrated cytotoxic effects on the MDA-MDB231 human breast cancer cells and the Caco-2 colorectal adenocarcinoma cell. ^7^ The study confirmed that potential bioactivity was present between the sponge-associated actinobacterium *Streptomyces* and its sponge host.

Molecular identification using the 16S rRNA gene sequence revealed that the strain BTA 1-131 is 98% similar to *S. kunmingensis* NBRC 14463. ^7^ A study by Wei et al. (2017) ^8^ reported the potential of *S. kunmingensis* YIM 121234 *,* which originated from soil, to produce bioactive compounds with potent cytotoxic activity towards the MCF-7 human breast cancer cells. Moreover, another reported strain, *S. kunmingensis* MR03, originated from marine sediment and showed its potential to synthesise cadmium sulphide nanoparticles that were active against biofilm-forming bacteria such as *S. aureus, E. coli, P. aeruginosa,* and *Enterecoccus* spp. ^9^

The diversity of *Streptomyces* and their novel compounds originating from marine biospheres remains less explored. ^10^ Indeed, the analysis of secondary metabolite BGCs demonstrated by Almeida et al. (2019) ^11^ suggests that while the marine isolates share a close genomic relationship with their terrestrial counterparts, they may have the capacity to produce distinct compounds. Considering the atypical origin of the culturable sponge symbiont *Streptomyces* sp. BTA 1-131, similarity of the strain to known *Streptomyces* (≤ 98.7%), and our intention to further evaluate its biosynthetic capabilities, we sequenced the whole genome of *Streptomyces* sp. BTA 1-131, using a de novo approach based on the hybrid Illumina and Nanopore platform. This has allowed us to generate a complete chromosome sequence. To the best of our knowledge, this is the first study to publish the complete genome of a sponge-associated *Streptomyces* strain from the marine sponge *M. sarasinorum* of Indonesian origin. The reported data provides the basis for further analysis to explore the strain as a putative new *Streptomyces* species and reveals the biosynthetic capabilities of its bioactive compounds.

## Methods

### Bacterial isolation and culture condition

*Streptomyces* sp. BTA 1-131 was previously isolated from marine sponge *M. sarasinorum* in December 2015. The sponge was collected in Tanjung Batu Angus, Lembeh Strait, Bitung, North Sulawesi, Indonesia (1.50572° N, 125.24541° E; depth 13 m) as described in the previous study. ^6^ The strain BTA 1-131 was previously identified by sequencing the 16S rRNA gene and is closely related to soil-derived *S. kunmingensis* NBRC 14463. ^7^ The nucleotide sequence of the 16S rRNA was deposited in the NCBI GenBank database under accession number MT280129, and the cultivable isolate was deposited in the Indonesian Culture Collection BRIN (InaCC) under accession number A1205. ^7^ In this study, the strain BTA 1-131 was revived from a glycerol stock stored at –80 °C. Prior to genomic DNA extraction, the strain BTA 1-131 was grown on an International Streptomyces Project (ISP)-recommended medium, ISP2 agar medium, at 28 °C, for 14 days. The ISP2 medium contains yeast extract (BD Difco, USA; 4 g L ^−1^), malt extract (BD Difco, USA; 10 g L ^−1^), and dextrose (BD Difco, USA; 4 g L ^−1^). The medium was prepared in 1 L of seawater with an addition of bacteriological agar (BD Difco, USA; 20 g L ^−1^).

### DNA extraction and sequencing

Genomic DNA was extracted from *Streptomyces* sp. BTA 1-131 colonies grown on ISP2 medium agar plate using the Quick-DNA ^TM^ HMW MagBead kit according to the manufacturer’s instructions (Zymoresearch, USA). The integrity of the extracted genomic DNA was assessed by gel electrophoresis (0.8% tris borate-EDTA agarose w/v), while its quality and quantity were analysed by a NanoDrop spectrophotometer (Thermo Fischer Scientific, USA) and a Qubit 4 fluorometer (Thermo Fischer Scientific, USA) using the Qubit ^TM^ dsDNA High Sensitivity assay kit (Thermo Fischer Scientific, USA), respectively. Moreover, total gDNA was used for the input of library preparation for both short read-based Illumina sequencing and long read-based ONT sequencing platforms.

To sequence the genome of the strain BTA 1-131 on the Illumina platform, the DNA library was prepared using the Illumina NextSeq500 system with the NextSeq 500/550 kit v2.5 (300 cycles) to generate 2 x 150 bp paired-end sequence reads following the manufacturer’s protocol (Illumina, USA). For the ONT platform, the DNA library was prepared for GridION ONT using the SQK-NBD114.24 ligation sequencing kit according to the manufacturer’s recommendation (Oxford Nanopore Technology, UK). In brief, total gDNA was repaired using an end prep enzyme mix, generating DNA with 5’ phosphorylated, 3’ dA-tailed ends. Barcodes were ligated with an ONT-compatible adapter. The library was quantified with a Qubit Fluorometer before loading to the FLO-MIN114 flow cell. Sequencing control was conducted in MINKNOW v23.04.6, and the output raw reads were set to POD5 for a separate basecalling process.

### Data processing and hybrid assembly

*Illumina read processing*

Illumina-generated FASTQ files were initially processed using Fastp v0.24.0 ^12^ to assess read quality and remove low-quality bases (Data file 1). ^13^ Reads were filtered with the parameters *-q 20 -l 75 -c*, ensuring only high-quality reads were retained (Data file 2). ^14^ Unpaired reads (those lacking a mate from either R1 or R2) were kept using the flags *–unpaired1 sample_unpaired1.fastq.gz --unpaired2 sample_unpaired2.fastq.gz* and were included alongside paired reads for subsequent hybrid assembly with the ONT data.

*ONT read processing and hybrid assembly*

Raw POD5 files were basecalled at super accuracy using Dorado v0.8.2, generating a simplex BAM file. This BAM file was converted to FASTQ format using Samtools v1.21. ^15^ Read quality distributions were assessed with NanoPlot v1.44.0, ^16^ (Data file 1). ^13^

Hybrid assembly began by assembling ONT reads first using Flye v2.9.5, ^17^ (Data file 3). ^18^ Parameters included *--nano-hq -g 8m --asm-coverage 50*, reflecting an estimated 8 Mbp genome size and a coverage limit set to 50X. Assembly graphs were visualised with Bandage v0.9.0, ^19^ (Data file 4). ^20^ Moreover, to refine the ONT-based assembly, Medaka v2.0.1 (Medaka consensus) was applied with recommended settings optimised for v14 reagent kit reads. A final hybrid assembly was then generated by polishing the ONT assembly with the high-quality Illumina reads using Pilon v1.24. ^21^ Three iterative polishing steps were performed; in the first iteration, the Illumina was aligned to the Medaka-polished ONT assembly using Bowtie v2.5.4, ^22^ and alignments were processed with Samtools v1.21. The resulting consensus contigs become assembly sources for the second iteration and continue until three iterations of polishing are reached. After these three polishing rounds constituted the final hybrid assembly for subsequent analyses.

*Assembly statistics and completeness*

Assembly metrics were obtained with the quality assessment tool for genome assembly (QUAST) v5.2.0, ^23^ reporting the number of contigs, N50, and GC content; gene predictions were disabled for this step (Data file 5). ^24^ Genome completeness was evaluated using the benchmarking universal single-copy orthologs (BUSCO) v5.8.2 ^25^ in *genome* mode, employing the *Streptomyces_odb12* lineage dataset (Data file 6). ^26^ The identified single-copy orthologs were visualised via the *generate_plot.py* script included with BUSCO. CheckM v1.2.3 ^27^ was used with the *lineage_wf* workflow to estimate completeness and contamination, summarised in a table of reported statistics *(qa)* (Data file 7). ^28^ Assemblies with >95% completeness and <5% contamination were considered high-quality.

### Bioinformatic analysis

*Genome visualisation and annotation*

Genome visualisation was performed using GenoVi v02.16, ^29^ which generated circular genome maps depicting sequence features, contig boundaries, and genomic architecture. Functional annotation of clusters of orthologous groups (COGs) categories was conducted using the integrated DeepNOG ^30^ within GenoVi, with gene sequences queried against the COG database to assign functional categories and identify orthologous proteins across genomes (Data file 8). ^31^ The resulting visualisations provided both an overview of overall genomic organisation and detailed functional annotation profiles for downstream analysis.

*TIR and plasmid identification*

To examine the linear inverted repeats on the strain BTA 1-131 chromosome, a basic local alignment search tool (BLAST) was conducted using the parameters described previously by Jørgensen et al. (2024). ^32^ While putative plasmid contigs were identified with PLASme v1.1 ^33^ using the default database and the “balance” mode parameter. All other settings were kept at default values. Any candidate plasmid contigs were verified by examining the assembly graph in Bandage to confirm their circular topology or unique structural features.

*Taxonomic inference and phylogenomic analysis*

Taxonomic assignment was carried out on the type (strain) genome server (TYGS) ^34^ by submitting the polished genome assembly (Data file 9). ^35^ Additional inferences were made using GTBD-Tk v2.1.0, ^36^ incorporating *Streptomyces kunmingensis strains* and the type strain *S. albus* NRRL B-181 (ASM72588v1), as well as some selected *Streptomyces* strains associated with marine sponges reported by Xu et al. (2019) ^37^ for comparative analysis. Moreover, *Deferribacter desulfuricans* SSM1 was chosen as an outgroup. The resulting genome BLAST distance phylogeny (GBDP) tree was visualised in iToL v7. ^38^ The TYGS webserver also provided a DNA-DNA hybridization (dDDH) score.

To assess the genomic similarity between the polished genome assembly of the strain BTA 1-131 and one of the reference *S. kunmingensis* DSM 41681 (the nearest strain based on the genome-scale GBDP tree), average nucleotide identity (ANI) was calculated using FastANI. ^39^ The reference genome sequence of *S. kunmingensis* DSM 41681 (ASM3561610v1) was obtained from publicly available genomic repositories, and the strain BTA 1-131 genome assembly was prepared using the following command: *FastANI -q assembly_genome.fasta -r reference_genome.fasta -o ani_output.txt*, where *-q* specifies the query genome (the polished genome assembly of the strain BTA 1-131), - *r* denotes the reference genome ( *S. kunmingensis* DSM 41681), and *-o* defines the output file storing ANI results. The ANI percentage from the analysis result was used to determine the genomic relatedness between the assembly and the reference strain. Results were interpreted based on established ANI thresholds (90–95%) for bacterial species delineation. ^39^

## Results

### Complete assembled genome description

The whole genome sequence of *Streptomyces* sp. BTA 1-131 was obtained from a total of 567,347 sequence reads from the ONT long-read assembly, followed by polishing with the Illumina short-read assembly. This hybrid assembly resulted in a 10,234,869 bp genome size with an N50 median of 9,496,435 bp and a GC content of 71.62%, consisting of one large chromosomal contig and three small contigs. Based on the CheckM analysis, the genome was 99.89% complete with a contamination level of 0.21%. In addition, the overall average coverage of the complete assembled genome was noted to be approximately 170.7X.

**Figure 1.**
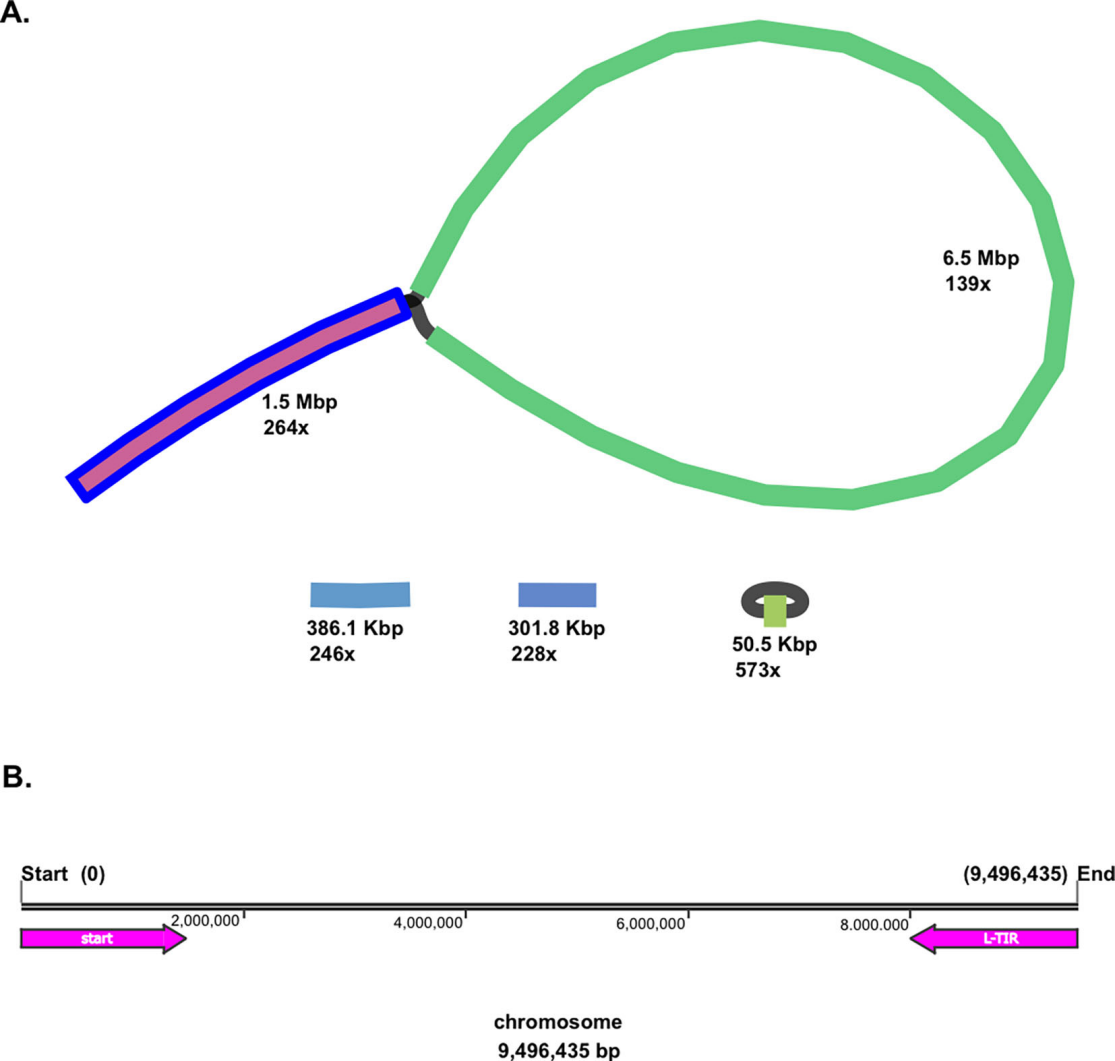
*Streptomyces* sp. BTA 1-131 assembly using Flye with high assembly depth. (A) Schematic representation of the *Streptomyces* sp. BTA 1-131 genome detected, adding up to 1.5 Mbp in length to chromosome size. In addition, a circular and two linear plasmids were detected from the strain BTA 1-131 assembly. (B) L-TIR is indicated with a reverse arrow (pink colour) from the end of chromosome (9,496,435–7,999,105; 1,497,324 bp).

Moreover, both linear and circular topologies were found in the genome of *Streptomyces* sp. BTA 1-131. The BLAST analysis revealed that a linear chromosomal genome of the strain BTA 1-131 contains a long-terminal inverted repeat (L-TIR) of up to 1.5 Mbp at the chromosome end (9,496,435–7,999,105; 1,497,730 bp), resulting in a loop-like feature ( Figure 1). Two additional small linear contigs, 386.1 Kbp and 301.8 Kbp in size, along with the smallest circular contig at 50.5 Kbp, were identified as plasmids. In total, there were 8,986 protein-coding genes (CDSs) with the average open reading frame (ORF) length of 995 bp. Within the genome *Streptomyces* sp. BTA 1-131, 21 rRNA and 92 tRNA operons were also predicted ( Table 1).

**Table 1.** Genomic features of *Streptomyces* sp. BTA 1-131, *S. kungnimensis* DSM 41681, and *S. albus* NRRL B-1811 ^T^.

	BTA 1-131	DSM 41681	NRRL B-1811 ^T^
Genome topology ^a^	linear	linear	linear
Contigs	4	379	104
Genome size (bp)	10,234,869	9,797,351	7,633,340
N50	9,496,435	57,076	245,715
GC content (%)	71.62	70.73	72.74
Protein coding genes (CDS)	8,986	8,633	6,405
rRNA genes	21	4	6
tRNA genes	92	83	81
Genes assigned to COG	87	78	36

^a^ Chromosome,

^T^ Type strain.

**Table 2.** Overview of data files/dataset generated in this study.

Label	Data	Type of data
Data file 1	Figshare: Quality distribution of the BTA1-131 reads from Illumina and ONT sequencing This data contains the raw data read quality score, GC content (%), and general statistics of the reads https://doi.org/10.6084/m9.figshare.24204633	Web report (HTML) Attribution: CC BY 4.0
Data file 2	Figshare: High-quality reads resulted from Illumina raw read filtering (Q > 20, length > 200) https://doi.org/10.6084/m9.figshare.24204720	Reads (FASTQ) Attribution: CC BY 4.0
Data file 3	Figshare: Draft assembly from ONT raw read This data contains fasta file, graph file, and assembly statistic. https://doi.org/10.6084/m9.figshare.30563048	Draft assembly (FASTA) Attribution: CC BY 4.0
Data file 4	Figshare: Topology hybrid assembly of *Streptomyces* sp. BTA 1-131 https://doi.org/10.6084/m9.figshare.30542942	Image (PNG) Attribution: CC BY 4.0
Data file 5	Figshare: Hybrid assembly statistic report https://doi.org/10.6084/m9.figshare.24204789	Report (ZIP) Attribution: CC BY 4.0
Data file 6	Figshare: Identification of BUSCO genes to assess genome completeness https://doi.org/10.6084/m9.figshare.24204855	Report (TXT) Attribution: CC BY 4.0
Data file 7	Figshare: Genome completeness and contamination assessment with CheckM https://doi.org/10.6084/m9.figshare.30563456	Report (TXT) Attribution: CC BY 4.0
Data file 8	Figshare: Genome visualisations including identified COGs https://doi.org/10.6084/m9.figshare.30564893	Report (PDF) Attribution: CC BY 4.0
Data file 9	Figshare: Identification for potential new species using TYGS https://doi.org/10.6084/m9.figshare.30563564	Report (PDF) Attribution: CC BY 4.0
Data set 1	NCBI GenBank: The nucleotide sequence of 16S rRNA gene of the strain BTA 1-131 NCBI accession number MT280129.1 https://www.ncbi.nlm.nih.gov/nuccore/MT280129	Contigs (FASTA)
Data set 2	The cultivable isolate: The Indonesian Culture Collection BRIN (InaCC), accession number A1205 This data contains a deposition data sheet of the isolate BTA 1-131 in InaCC https://hdl.handle.net/20.500.12690/RIN/UKGE4I	Report (PDF) Attribution: CC BY-NC-ND 4.0
Data set 3	Raw reads from Illumina and ONT sequencers This data contains raw reads deposited in the DNA Data Bank of Japan (DDBJ) and Indonesian national scientific repository (RIN Dataverse) DDBJ Bioproject: PRJDB16533 https://ddbj.nig.ac.jp/search/entry/bioproject/PRJDB16533 RIN Dataverse: https://hdl.handle.net/20.500.12690/RIN/UQP1WS	GenBank collection (FASTQ)

**Figure 2.**
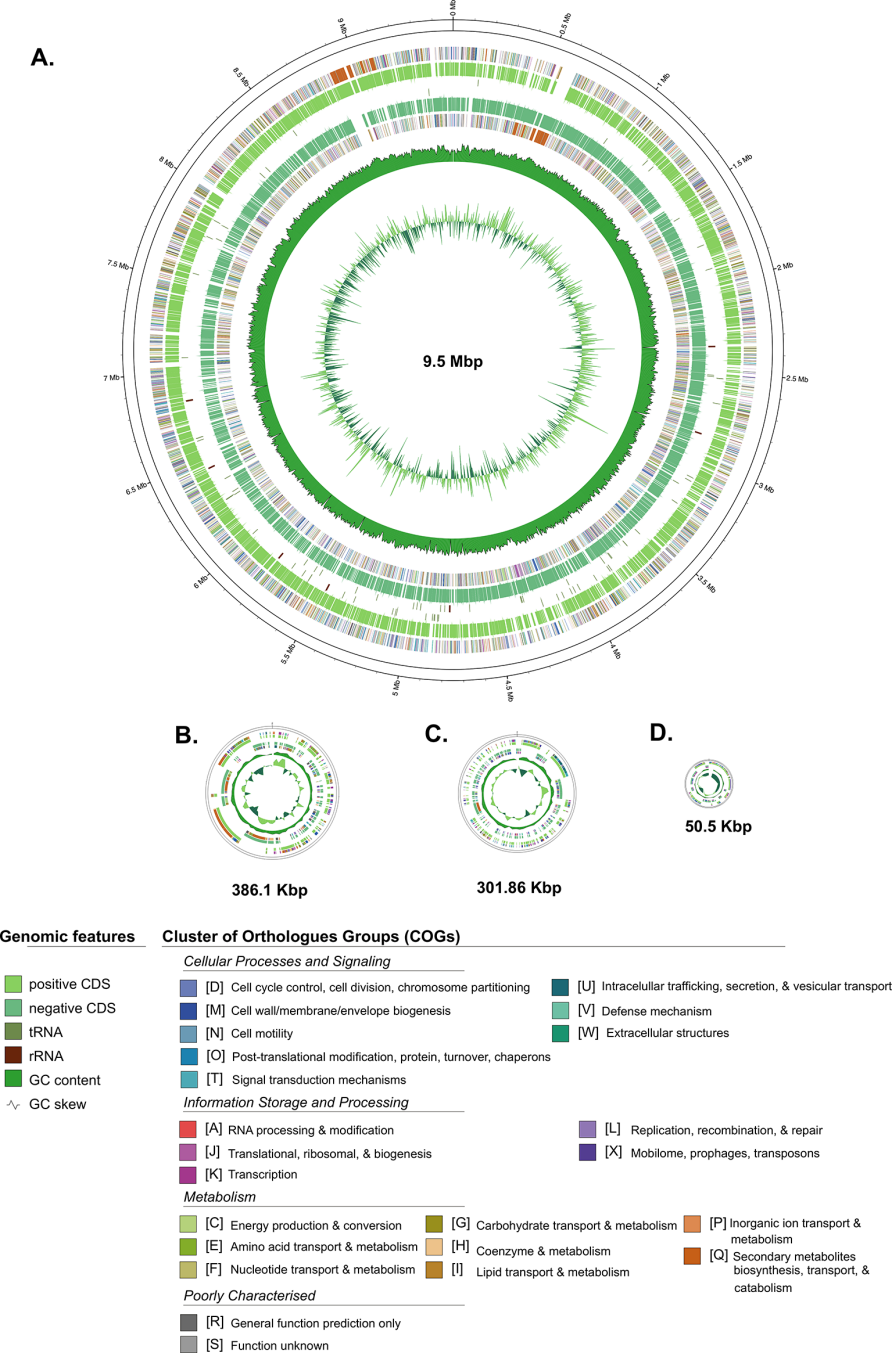
A circular map of each contig from the Streptomyces sp. BTA 1-131 assembly. Contig of L-TIR displayed together as one chromosome. Labelling from outside to the inside: contigs; COGs on the forward strand; CDS, rRNAs, and tRNAs on the forward strand; CDS, rRNAs, and tRNAs on the reverse strand; GC content; GC skew.

Furthermore, the general genome features of *Streptomyces* sp. BTA 1-131 are summarised in comparison to its closely related neighbour strain *S. kunmingensis* DSM 41681 and the type strain *S. albus* NRRL B-1811 in Table 1, while the schematic of circular maps of the genome, including the annotated COG categories, is presented in Figure 2.

### Phylogenomic analysis

**Figure 3.**
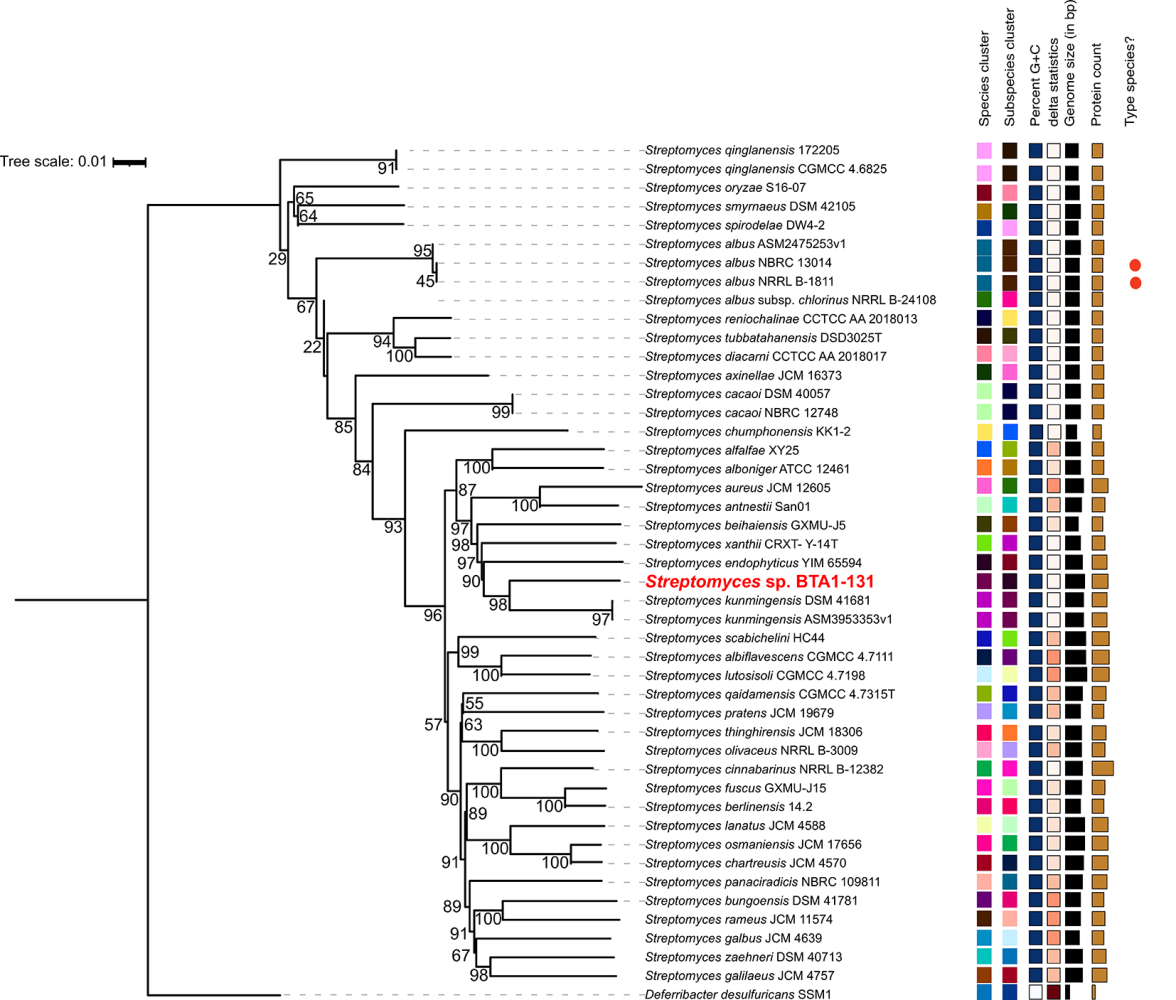
Phylogenomic tree construct based on whole genome sequence data of *Streptomyces* sp. BTA 1-131 and other *Streptomyces* strains.

As aforementioned, the strain BTA 1-131 was previously identified by sequencing the 16S rRNA gene and is closely related to *S. kunmingensis* NBRC 14463 with the sequence similarity of 98.4%. Indeed, the results of TYGS species identification suggested a potential novel *Streptomyces* species for the strain BTA 1-131, as indicated by the dDDH value below the threshold for classification as the same species as the nearest strain, *S. kunmingensis* DSM 41681 (<70%) ( Figure 3). Moreover, the ANI score between the BTA 1-131 genome assembly and *S. kunmingensis* DSM 41681 was 89.15%.

### Limitations

The main limitation in this study is that although the phylogenomic analysis showed a possibility of the strain BTA 1-131 as a new *Streptomyces* species, a follow-up taxonomical confirmation through polyphasic study is necessary to establish its species status. Additional taxonomical investigations, including chemotaxonomic, morphological, and biochemical characterisation, would be required to definitively confirm the novelty of this strain and validate its classification within the genus *Streptomyces.*

## Ethical considerations

Not applicable.

## Data Availability

The data generated from this study is available in Table 2. The sequence read archives are deposited in Genbank: 16S rRNA (Accession no. MT280129.1), ^40^ Illumina and ONT reads (Bioproject PRJDB16533). ^41^ The raw reads are also accessible in the Indonesian national scientific repository (RIN Dataverse): https://hdl.handle.net/20.500.12690/RIN/UQP1WS. The cultivable isolate was deposited in the Indonesian Culture Collection BRIN (InaCC) under accession number InaCC A1205DNA and is accessible in RIN Dataverse: https://hdl.handle.net/20.500.12690/RIN/UKGE4I. ^42^
