# Supplementary material for: The first whole-genome sequence of a prospective novel sponge-associated Streptomyces strain from Indonesia with a long 1.5 Mbp terminal inverted repeat
Source: F1000Res. 2026 Jan 24;14:1303. Originally published 2025 Nov 24. [Version 3] doi: 10.12688/f1000research.173356.3 (PMC12824479; doi:10.12688/f1000research.173356.3)
Supplement: Peer review history

# Reviewer report: Reviewer response for version 2

**DOI:** 10.5256/f1000research.195110.r450494

**Published:** 2026-01-21

**Authors:** Arzu Karahan

**Affiliations:** 1 Middle East Technical University, Erdemli-Mersin, Turkey

I don't have further comment; this version can be accepted for publication.

Are the datasets clearly presented in a usable and accessible format, and the assembly and annotation available in an appropriate subject-specific repository?

Partly

Are sufficient details of the sequencing and extraction, software used, and materials provided to allow replication by others?

Yes

Are the rationale for sequencing the genome and the species significance clearly described?

Yes

Are the protocols appropriate and is the work technically sound?

Yes

Reviewer Expertise:

tunicates, aging, whole body regeneration, transcriptomics, metagenomics.

I confirm that I have read this submission and believe that I have an appropriate level of expertise to confirm that it is of an acceptable scientific standard.

# Reviewer report: Reviewer response for version 1

**DOI:** 10.5256/f1000research.191165.r442272

**Published:** 2026-01-12

**Authors:** Matsapume Detcharoen

**Affiliations:** 1 Prince of Songkla University, Songkhla, Thailand

This Genome Note reports a high-quality hybrid Illumina and Oxford Nanopore whole-genome assembly of Streptomyces sp. BTA 1-131, a sponge-associated strain isolated from the Indonesian marine sponge Melophlus sarasinorum. The genome is about 10 Mbp with an unusually long 1.5 Mbp terminal inverted repeat and plasmids.

 Define “ONT” at first use.

 Specify the end-prep chemistry and kits used prior to ONT library construction.

 Explain why barcodes were used, identify the barcode kit, and clarify whether multiplexing was performed.

 Report how many libraries were constructed, the number and type of flow cells used, and the duration of the sequencing run.

 Clearly describe ONT data quality control, including read filtering, trimming, and summary statistics such as read N50 and quality scores.

 The rationale for the estimated genome size used during Flye assembly should be explained. Given the unusually long 1.5 Mbp terminal inverted repeat (TIR), stronger validation is warranted, such as read-level support across TIR boundaries or additional assembly diagnostics, to confirm that this feature is not an assembly artifact.

 The manuscript inconsistently refers to the reference strain as “NRRL B-181” and “NRRL B-1811.” This must be corrected throughout.

 Although BUSCO and CheckM analyses were performed, BUSCO results are not clearly summarized in the main text.

 Data set 2 and 3 are not yet public.

 Clarify inconsistencies in Illumina read filtering thresholds between the Methods section and Table 2.

 Correct typographical errors, including the misspelling of Streptomyces kunmingensis in Table 1.

 Figure 3 requires a scale bar and an explanation of color coding.

 The manuscript does not clearly describe how the phylogenetic tree shown in Figure 3 was reconstructed.

 a brief summary of predicted secondary metabolite biosynthetic gene clusters would strengthen the manuscript

Are the datasets clearly presented in a usable and accessible format, and the assembly and annotation available in an appropriate subject-specific repository?

Yes

Are sufficient details of the sequencing and extraction, software used, and materials provided to allow replication by others?

Yes

Are the rationale for sequencing the genome and the species significance clearly described?

Partly

Are the protocols appropriate and is the work technically sound?

Partly

Reviewer Expertise:

Genomics, molecular ecology

I confirm that I have read this submission and believe that I have an appropriate level of expertise to confirm that it is of an acceptable scientific standard.

# Response

**Published:** 2026-01-15

**Authors:** Linda Sukmarini

**Affiliations:** RC Applied Microbiology, National Research and Innovation (BRIN), Republic of Indonesia, Bogor, West Java, Indonesia

Thank you Reviewer for the critical input to our manuscript. Below are our responds to the comments:

 We have updated the manuscript to the third version (v3), however, we are waiting for the second version (v2) to be published first. We have already submitted the v2 before receiving your comments. 

 1. We have updated manuscript (v3) to define "ONT" on first use

 2. The End-prep chemistry have been added to the method section

 3. The sequencing process of BTA1-131 with ONT platform was joined with other projects (multiplexing), therefore barcodes were used. The barcode kit already mentioned in the methods section (SQK-NBD114.24).

 4. Only one library was generated. The flowcell used already mentioned in the methods section (FLO-MIN114). The sequencing run was executed for 72 hours

 5. The summary statistics for ONT quality control have been revised in the manuscript. We did not perform filtering or trimming, since the Flye assembler need to use all data for the assembly.

 6. Genome size estimation during assembly process was calculated from the average of Streptomyces spp. genome size. We believe that the high coverage number from the assembly result, support the finding of TIR region

 7. We have updated the manuscript (v3) for "NRRL B-1811" consistently written

 8. We have updated manuscript (v3) to include BUSCO result after CheckM result.

 9.  For access to the dataset 2 and 3 of RIN Dataverse, the project has been published, however access to the raw data is available by request. 

 For dataset 3, access to the DDBJ bioproject PRJDB16533 will be released after manuscript been published. The same data are mirrored in the RIN Dataverse (dataset 3) and available upon request in the system.

 10. We have updated the manuscript (v3) to correct the inconsistency for Illumina filtering thresold.

 11. The scale bar revision have been submitted alongside v2 manuscript. The color coding was default to TYGS result, such as the species cluster, subspecies cluster, and other parameters which is described in the TYGS paper (doi.org/10.1038/s41467-019-10210-3). We do not receive detailed explanation from the TYGS results (Data File 9)

 12. The construction of Phylogenetic tree is already mentioned in the methods section, under " *Taxonomic inference and phylogenomic analysis*". This tree was part of the TYGS results, we just better visualized using iTOL (also described already)

 13. As mentioned earlier in the introduction, this manuscript focuses on the finding of the complete genome of a sponge-associated *Streptomyces* strain from the marine sponge *M. sarasinorum* of Indonesian origin. We highlight the TIR feature and species delineation. The secondary metabolite analysis is already prepared in the follow up publication.

# Reviewer report: Reviewer response for version 1

**DOI:** 10.5256/f1000research.191165.r436015

**Published:** 2025-12-27

**Authors:** Arzu Karahan

**Affiliations:** 1 Middle East Technical University, Erdemli-Mersin, Turkey

In the article, Streptomyces sp. BTA 1-131, isolated from the rare marine sponge Melophlus sarasinorum, is sequenced using a hybrid Illumina-Nanopore approach, resulting in a high-quality genome assembly. Based on the phylogenomic analyses, the strain is suggested to represent a new species.

 Overall, this is a well-prepared manuscript; however, a few issues need to be addressed:

 - The scale bar in Figure 3 is missing.

 - Datasets 2 and 3 require a password for access, preventing verification of the associated data.

Are the datasets clearly presented in a usable and accessible format, and the assembly and annotation available in an appropriate subject-specific repository?

Partly

Are sufficient details of the sequencing and extraction, software used, and materials provided to allow replication by others?

Yes

Are the rationale for sequencing the genome and the species significance clearly described?

Yes

Are the protocols appropriate and is the work technically sound?

Yes

Reviewer Expertise:

tunicates, aging, whole body regeneration, transcriptomics, metagenomics.

I confirm that I have read this submission and believe that I have an appropriate level of expertise to confirm that it is of an acceptable scientific standard, however I have significant reservations, as outlined above.

# Response

**Published:** 2026-01-09

**Authors:** Linda Sukmarini

**Affiliations:** RC Applied Microbiology, National Research and Innovation (BRIN), Republic of Indonesia, Bogor, West Java, Indonesia

Thank you reviewer for your kind report. To answer your questions:

 1. We have revised the phylogenetic tree figure to include the tree scale. The figure will be included in the second version of article

 2. For access to the dataset 2 and 3 of RIN Dataverse, the project has been published, however access to the raw data is available by request. 

 For dataset 3, access to the DDBJ bioproject PRJDB16533 will be released after manuscript been published. The same data are mirrored in the RIN Dataverse (dataset 3) and available upon request in the system.
